# Association of iron metabolism with disease severity in acute Puumala hantavirus infection

**DOI:** 10.1186/s12882-025-04609-y

**Published:** 2025-12-19

**Authors:** Johanna Tietäväinen, Tuula Outinen, Ulla Nivukoski, Outi Laine, Satu Mäkelä, Heini Huhtala, Onni Niemelä, Ilkka Pörsti, Antti Vaheri, Jukka Mustonen

**Affiliations:** 1 Department of Internal Medicine, Tampere University Hospital, Wellbeing Services County of Pirkanmaa, Tampere, Finland; 2 Faculty of Medicine and Health Technology, Tampere University, Tampere, Finland; 3 Medical Research Unit, Seinäjoki Central Hospital, Wellbeing Services County of South Ostrobothnia, Seinäjoki, Finland; 4 Faculty of Social Sciences, Tampere University, Tampere, Finland; 5 Department of Virology, Faculty of Medicine, University of Helsinki, Helsinki, Finland; 6 Tampere University Hospital, P.O. Box 2000, Tampere, 33521 Finland

**Keywords:** Puumala hantavirus, Acute kidney injury, Iron, Ferritin, Hepcidin, Neutrophil-gelatinase associated lipocalin

## Abstract

**Background:**

Serum ferritin is associated with disease severity in haemorrhagic fevers caused by various viruses, while iron has been implicated in the development of acute kidney injury (AKI). Puumala hantavirus causes a haemorrhagic fever with renal syndrome with acute kidney injury (AKI), thrombocytopenia and increased vascular leakage. Histologically acute haemorrhagic tubulointerstitial nephritis is detected. Degrading red blood cells in the kidney are an abundant source of iron and ferritin. Free iron causes oxidative stress. We aimed to evaluate the role of iron metabolism in haemorrhagic fever with renal syndrome caused by Puumala hantavirus.

**Methods:**

Serum samples from 58 patients in the acute phase and from 51/58 (88%) in the convalescence phase were analysed for variables of iron metabolism and their relationship to the disease severity of acute PUUV infection.

**Results:**

During the acute phase, the median concentrations of serum ferritin (1136 vs. 225 µg/L, *p* < 0.001), hepcidin (386 vs. 51 ng/mL, *p* < 0.001), and neutrophil gelatinase-associated lipocalin (NGAL) (85 vs. 45 ng/mL, *p* < 0.001) were elevated, while the median concentrations of serum iron (8.4 vs. 16.2 µmol/L, *p* < 0.001) and transferrin (1.80 vs. 2.6 g/L, *p* < 0.001) were reduced compared with the convalescence phase. Serum ferritin, hepcidin, and NGAL levels correlated directly with blood leukocyte count (*r* = 0.307, *p* = 0.019; *r* = 0.318, *p* = 0.015: *r* = 0.761, *p* < 0.001), while the relationship between leukocytes and transferrin was inverse (*r*=-0.314, *p* = 0.016). Serum ferritin and hepcidin showed an inverse correlation with the minimum platelet count (*r*=-0.260, *p* = 0.048, *r*=-0.296, *p* = 0.024, respectively). However, serum ferritin, hepcidin and transferrin did not correlate with serum creatinine or NGAL.

**Conclusion:**

Increased serum ferritin and hepcidin levels, as well as decreased transferrin levels, were associated with high leukocyte counts and low platelet counts during acute PUUV infection. However, parameters of iron metabolism were not associated with the severity of AKI in this infection.

## Introduction

Puumala hantavirus (PUUV) is a member of the *Orthohantavirus* genus in the *Hantaviridae* family, order *Bunyavirales.* It is carried by the bank vole (*Myodes glareolus*) and causes a mild form of haemorrhagic fever with renal syndrome (HFRS), which has the highest incidence in the world in Finland [1, 2]. PUUV-induced hemorrhagic fever with renal syndrome (HFRS) is characterized by acute kidney injury (AKI), thrombocytopenia, and increased capillary permeability, which are reflected by the peak serum creatinine concentration, the lowest platelet count, and the lowest blood pressure recorded during hospitalization. The duration of hospital stay serves as an indicator of overall disease severity. In severe cases, patients may present with clinical shock. About 6% of the hospitalized patients need transient dialysis treatment. Bleeding diathesis is rare and case fatality in Finland is less than 0.1% [1, 3].

Serum ferritin is implicated in the severity of haemorrhagic fevers caused by different viruses [4–8]. In HFRS caused by Hantaan virus, high ferritin was found to predict disease severity and mortality [4]. Ferritin also associated with disease severity in dengue fever, Crimean-Congo haemorrhagic fever, and in severe fever with thrombocytopenia [5, 7–9]. 

Iron is crucial for DNA synthesis and ATP generation and therefore essential also for viral replication. Viruses can alter iron metabolism to enhance replication and the host immune system limits the availability of iron during infection [10, 11]. Infection and inflammation are often followed by anemia. Free iron can produce reactive oxygen species and damage cellular structures. The kidney proximal tubules and medullary thick ascending limb cells are especially sensitive to oxidative stress due to high metabolic activity [12].

PUUV causes an acute haemorrhagic tubulointerstitial nephritis and as a maker of proximal tubular damage, patients with severe disease have glucosuria in addition to albuminuria and haematuria [13–16]. Iron is released from degrading red blood cells. The role of iron and changes in proteins involved in iron metabolism have been linked to the development of both AKI and chronic kidney disease (CKD) [17–19], while urine iron metabolites have been proposed as possible biomarkers for kidney diseases [20]. However, there is limited information available on the relationship between variables of iron metabolism during hantavirus infections. We aimed to evaluate the variables of iron balance during the acute and convalescence phases of PUUV infection and their association with the severity of the infection and AKI.

## Materials and methods

### Subjects

The study cohort included 58 patients treated at Tampere University Hospital, Finland, for serologically confirmed acute PUUV infection between 2001 and 2017. The patients arrived at the hospital median day four (Q1-Q3; 3–5) after the onset of fever. A detailed medical history was obtained for all patients, and each patient underwent a thorough clinical examination. Blood pressure, heart rate, weight, and daily urine output were measured during hospital care. Changes in body weight were recorded in kilograms rather than as percentage changes, which would have better accounted for differences in body composition. Laboratory samples were taken daily during the acute infection.

Out of 58 patients, 21 (36%) had one or more pre-existing conditions, including hypertension (*n* = 9), hypercholesterolemia (*n* = 3), cardiac arrhythmia (*n* = 3), coronary heart disease (*n* = 2), inflammatory bowel disease (2), prostate hyperplasia (*n* = 2), asthma (*n* = 2), coeliac disease (*n* = 1), type 2 diabetes (*n* = 1), psoriasis (*n* = 1), sarcoidosis (*n* = 1), and a history of surgically treated skin malignancy (*n* = 1). None of the patients had a known CKD before the PUUV infection.

### Definition of AKI

According to Kidney Disease: Improving Global Outcomes (KDIGO) criteria, AKI is diagnosed based on an increase in serum creatinine (S-creatinine) from baseline and/or reduced urine output. In this study, all patients were treated in general hospital wards rather than in the ICU. While S-creatinine and daily urine output were monitored, hourly diuresis was not recorded. Furthermore, baseline S-creatinine levels prior to the Puumala virus (PUUV) infection were unavailable, making it challenging to apply the KDIGO criteria accurately. Therefore, the study included all patients with confirmed PUUV infection, independent of S-creatinine levels or urine output. All analyses were conducted using the maximum S-creatinine value recorded during hospitalization as a continuous variable.

All patients provided a written informed consent, and the study and its’ extensions were conducted according to the guidelines of the Declaration of Helsinki and approved by the Ethics Committee of the Tampere University Hospital (study codes *99256*,* R15007 and R22032*).

### Laboratory methods

The diagnosis of PUUV infection was made by detecting the typical granular staining pattern in immunofluorescence assays, low avidity of IgG antibodies to PUUV, and/or by detecting PUUV IgM antibodies using an in-house enzyme-linked immunosorbent assay based on a recombinant antigen. The development and the use of the above diagnostic methods have been described elsewhere [21]. 

Serum creatinine, plasma and urine albumin and C-reactive protein concentrations were analysed using a Cobas Integra analyser for clinical chemistry and immunochemistry testing (F. Hoffmann-La Roche Ltd., Basel, Switzerland). Blood cell counts were determined by automated haematological cell counters (Bayer Diagnostics, Elkhart, IN, USA).

Serum iron concentrations were analysed photometrically on a Cobas 8000 c702 chemistry analyser (Roche Diagnostics, Mannheim, Germany), and serum ferritin was measured by electrochemiluminescence immunoassay (ECLIA on Cobas 8000 e801 immunoassay analyser, Roche Diagnostics, Mannheim, Germany). Serum haptoglobin, transferrin, and transferrin receptor concentrations were measured by immunoturbidimetric methods on the Cobas 8000 c502 chemistry analyser (Roche Diagnostics, Mannheim, Germany). Serum lactate dehydrogenase (LDH) was analysed photometrically on the Cobas 8000 c702 chemistry analyser (Roche Diagnostics, Mannheim, Germany), in accordance with the recommendations of the International Federation of Clinical Chemistry and Laboratory Medicine.

Serum neutrophil gelatinase-associated lipocalin (NGAL) was analysed using the Human Lipocalin-2 ELISA kit (BioVendor, Brno, Czech Republic). Serum hepcidin was analysed using the Human Hepc (Hepcidin) ELISA kit (Elabscience, Texas, USA). All measurements were performed in an SFS-EN ISO 15189:2013 accredited laboratory.

The laboratory analyses were carried out a median of 7 days (Q1-Q3; 6–8) after the onset of fever (acute phase), and again a median of 41 (Q1-Q3; 37–46) days after the onset of fever (convalescence phase). The data for serum iron parameters were available from 58 patients in the acute phase and from 51/58 (88%) patients in the convalescence phase. Serum creatinine, plasma C-reactive protein (CRP), and blood cell counts, including red blood cell indices, leukocytes, and platelets, were measured in all study subjects. Information on body weight changes during the hospital stay, reflecting fluid accumulation during AKI and capillary leakage during inflammation, and the length of hospital stay were available for all patients.

### Statistical analysis

The data are presented as medians and quartiles for continuous variables and numbers and percentages for categorical variables. Consecutive values were compared using Wilcoxon signed ranks test. The Spearman correlation (r_S_) was used to study the relationship between the different variables. All analyses were performed using IBM SPSS Statistics version 28 (IBM, Armonk, NY, USA).

## Results

The median age of the patients was 44 years and 41/58 (71%) were men. The main clinical and laboratory characteristics of the patients with acute PUUV infection are presented in Table 1. Out of 58 patients five needed transient dialysis treatment. None of the patients received iron supplementation and none received blood transfusions during hospital treatment.

**Table 1:** Clinical and laboratory findings of 58 patients with acute PUUV infection

Variable	Median/number	Q1-Q3/%	Range
Age (years)	44	34–56	22–77
Male/female	41/17	71/29	
Body mass index (kg/m^2^)	25	22–28	20–35
Shock (yes/no)*	1/57	2/98	
Min systolic BP (mmHg)	113	104–133	60–162
Min diastolic BP (mmHg)	70	64–80	46–100
Weight change (kg)^†^	3.4	1.3–5.4	0.1–12.5
Length of hospital stay (days)	7	5–8	3–15
Haemoglobin min (g/L)	124	112–133	68–175
Haemoglobin max (g/L)	145	134–164	86–243
Leukocytes max (×10^9^/ml)	10.2	8.1–13.1	3.8–50.3
Thrombocytes min (×10^9^/ml)	69	44–101	3-389
CRP max (mg/L)	69	42–104	6-305
Creatinine max (µmol/L)	223	120–462	51-1645

*Systolic blood pressure < 90 mmHg and clinical signs and symptoms of a shock

^†^Weight change during hospital stay reflecting fluid retention during acute PUUV infection. Abbreviations: PUUV, Puumala hantavirus; Q, quartile; BP, blood pressure; CRP, C-reactive protein. The results are presented as median and interquartile ranges for continuous variables and numbers and percentages for categorical values

Serum iron, ferritin, transferrin, transferrin iron saturation (TSAT), hepcidin, LDH, haptoglobin and NGAL were chosen to reflect the body’s iron stores and iron metabolism. NGAL is also known as a biomarker of AKI.

The concentrations of serum iron, transferrin, TSAT, and transferrin receptor were significantly lower during the acute phase compared to the convalescence phase (Table 2). The concentrations of all other variables reflecting iron metabolism and/or kidney function were significantly higher during the acute phase (Table 2).

**Table 2:** Comparison of serum laboratory findings in patients with PUUV infection in the acute phase (median 7 days after the onset of fever) and the convalescence phase (median 41 days after the onset of fever)

	Acute (*n* = 51)			Convalescence (*n* = 51)			
Variable	Median	Range	Q1-Q3	Median	Range	Q1-Q3	*p*-value
Iron (9–34 µmol/L)	8.4	2.2–22.3	6.0-12.4	16.2	3.7–35.5	13.5–19.8	**< 0.001**
Transferrin (2.0–3.6 g/L)	1.8	1.2–3.2	1.5-2.0	2.6	1.9–4.1	2.3–2.8	**< 0.001**
TSAT % (15–50%)	18.4	4.8–62.8	13.2–27.1	24.7	6.4–46.7	19.5–28.5	**0.012**
TfR (1.9–4.4 mg/L (F), 2.2-5.0 mg/L (M))	2.6	1.8–4.3	2.2–3.1	3.3	1.9-5.0	2.9-4.0	**< 0.001**
Ferritin (13–150 µg/L (F), 30–400 µg/L (M))	1136	17-14620	693–1505	225	11–753	109–369	**< 0.001**
Haptoglobin (0.29–2.0 g/L)	2.3	1.0-3.9	1.8–2.5	1.4	0.4–3.1	1.0-1.7	**< 0.001**
LDH (105–205 U/L)	238	64–409	198–291	163	93–229	155–182	**< 0.001**
NGAL (ng/mL)	85	27–300	64–134	45	27–147	39–62	**< 0.001**
Hepcidin (ng/mL)	386	27-5800	280–542	51	2-264	27–93	**< 0.001**

PUUV, Puumala hantavirus; Q, quartile, TfR, transferrin receptor; TSAT, transferrin iron saturation; F, female; M, male; LDH, lactate dehydrogenase; NGAL, neutrophil gelatinase-associated lipocalin; p-values refer to Wilcoxon signed-rank test

The minimum haemoglobin concentration during the acute phase correlated directly with acute phase transferrin and negatively with NGAL levels (as well as with maximum serum creatinine, r_S_ = -0.399, *p* = 0.002). Moreover, the minimum haemoglobin concentration during the acute phase correlated directly with serum iron, ferritin, and hepcidin concentrations during the convalescence phase, and negatively with the transferrin receptor concentration. However, it did not correlate with the same parameters during the acute phase (Table 3).

**Table 3:** Spearman correlation (r_S_) coefficients of acute phase minimum haemoglobin with parameters of iron metabolism in the acute phase and the convalescence phase of PUUV infection

Serum variables (normal range)	*r*_S_ for acute phase haemoglobin minimum with acute phase variable	*p*-value	*r*_S_ for acute phase haemoglobin minimum with convalescence phase variable	*p*-value
Iron (9–34 µmol/L)	0.149	0.263	0.350	**0.012**
Transferrin (2.0–3.6 g/L)	0.454	**< 0.001**	0.106	0.459
TSAT % (15–50%)	-0.013	0.920	0.215	0.130
TfR (1.9–4.4 mg/L (F), 2.2-5.0 mg/L (M))	0.173	0.194	-0.312	**0.026**
Ferritin (13–150 µg/L (F), 30–400 µg/L (M))	-0.010	0.943	0.322	**0.023**
Haptoglobin (0.29–2.0 g/L)	0.041	0.761	0.033	0.819
LDH (105–205 U/L)	-0.062	0.641	-0.067	0.642
NGAL (ng/ml)	-0.337	**0.010**	0.224	0.115
Hepcidin (ng/ml)	-0.052	0.696	0.303	**0.031**

PUUV, Puumala hantavirus; TSAT, transferrin iron saturation; TfR, transferrin receptor; F, female; M, male; LDH, lactate dehydrogenase; NGAL, neutrophil gelatinase-associated lipocalin

Table 4 presents the Spearman correlations of acute phase serum iron parameters with indices of disease severity. Excluding serum iron concentration, several variables of iron metabolism correlated with the inflammatory markers maximum leukocyte count and CRP, as well as with the minimum platelet count. However, the serum iron parameters transferrin, ferritin, and hepcidin did not correlate with the acute phase maximum creatinine concentration (Table 4). Transferrin, ferritin and hepcidin did not correlate with the acute phase NGAL either (data not shown). The need for dialysis was not associated with the levels of acute phase serum iron, transferrin, ferritin or hepcidin concentrations (data not shown). As expected, serum creatinine and NGAL showed a strong direct correlation (r_S_ = 0.761, *p* < 0.001) [22].

**Table 4:** Spearman correlation coefficients (r_S_) of acute phase serum iron parameters with disease severity markers in 58 patients with acute PUUV infection

		Fe	Transferrin	Ferritin	Hepcidin	Haptoglobin	LDH	NGAL
Creatinine max	r_S_	-0.172	-0.177	-0.012	0.094	0.028	0.332*	0.761**
	p-value	0.197	0.185	0.928	0.483	0.835	**0.011**	**< 0.001**
Leukocyte count max	r_S_	-0.178	-0.314*	0.307*	0.318*	0.051	0.434**	0.451**
	p-value	0.182	**0.016**	**0.019**	**0.015**	0.705	**0.001**	**< 0.001**
Platelet count min	r_S_	-0.230	0.271*	-0.260*	-0.296*	0.283*	-0.313*	0.175
	p-value	0.083	**0.040**	**0.048**	**0.024**	**0.032**	**0.017**	0.188
CRP max	r_S_	-0.180	-0.365**	0.321*	0.273*	0.309*	-0.279*	-0.021
	p-value	0.177	**0.005**	**0.014**	**0.038**	**0.018**	**0.034**	0.878
Weight change	r_S_	-0.106	-0.481**	0.331*	0.377**	-0.301*	0.331*	0.370**
	p-value	0.450	**< 0.001**	**0.016**	**0.005**	**0.028**	**0.016**	**0.006**
Systolic BP min	r_S_	-0.109	0.289*	-0.094	0.004	0.160	0.160	0.086
	p-value	0.415	**0.028**	0.482	0.974	0.230	0.230	0.520
Length of hospital stay	r_S_	-0.067	-0.428**	0.276*	0.314*	-0.030	0.214	0.251
	p-value	0.615	**0.001**	**0.036**	**0.016**	0.821	0.107	0.058

PUUV, Puumala hantavirus; Fe, iron; LDH, lactate dehydrogenase; NGAL, neutrophil gelatinase-associated lipocalin; CRP, C-reactive protein; BP, blood pressure; weight change during hospital treatment reflects the fluid retention during acute PUUV infection

Serum creatinine correlated with the change in weight during hospital care (r_S_ = 0.535, *p* < 0.001). In addition, all iron metabolism parameters, with the exception of serum iron concentration, were correlated with changes in body weight during hospitalization. Only transferrin, but not the other parameters of iron metabolism, correlated with the minimum systolic blood pressure. Serum transferrin correlated inversely, and serum ferritin and hepcidin correlated directly, with the length of hospital stay (Table 4). Unlike serum creatinine (r_S_ = 0.355, *p* = 0.006), NGAL did not correlate with the length of hospital stay.

In addition, the acute phase serum creatinine maximum correlated inversely with the convalescence phase iron (*r* = -0.427, *p* = 0.002) and transferrin (*r* = -0.363, *p* = 0.009) concentrations, while the acute phase NGAL correlated inversely with acute (*r* = -0.306, *p* = 0.019) and convalescence (*r* = -0.466, *p* < 0.001) phase serum iron concentrations and with convalescence phase serum transferrin concentration (*r* = -0.319, *p* = 0.023). Acute phase minimum platelet count correlated positively (*r* = 0.384, *p* = 0.005) and maximum CRP level negatively (*r* = -0.342, *p* = 0.014) with convalescence phase NGAL.

## Discussion

In this study, we found that several parameters reflecting iron metabolism changed significantly during acute PUUV infection, with normalisation of the values in the convalescence phase. Serum ferritin and hepcidin correlated directly with markers of disease severity, including inflammation, thrombocytopenia, and the length of hospital stay, all of which reflect the overall severity of the disease. However, ferritin and hepcidin were not associated with the severity of AKI.

Iron is an essential trace element for all living organisms [10]. It is a key constituent of haemoglobin and myoglobin. Small amounts of iron are incorporated into enzymes and other proteins, especially in the mitochondria, where it plays a crucial role in energy production through oxidative phosphorylation. Iron has the ability to produce reactive oxygen species and cause toxic effects, which is why its metabolism is tightly regulated.

In serum, iron is transferred by its carrier protein transferrin. Non-heme iron is incorporated into ferritin, which is found in all cell types. Ferritin-bound iron is stored in the bone marrow and the reticuloendothelial system and distributed to tissues as needed. Systemically, iron metabolism is regulated by hepcidin, which binds to the only known cellular iron exporter ferroportin, guiding ferroportin to degradation in the lysosomes. This limits the absorption of iron into enterocytes and into the plasma membrane of macrophages. The production of hepcidin is influenced by iron stores and by the inflammatory status.

As invading microbes also need iron, the availability of iron is limited during infection [10, 11]. Several components of iron metabolism are acute-phase proteins, the serum concentrations of which change during inflammation. When leukocytes secrete cytokines, the liver responds by producing several acute-phase proteins. Ferritin, hepcidin, and haptoglobin are acute-phase proteins, the concentrations of which increase during inflammation, while that of transferrin decreases [10, 11], as was also shown in the present study in patients with acute PUUV infection. Some microbes can even degrade red blood cells and separate iron from heme. Haptoglobin and hemopexin compete with the microbes for binding free haemoglobin. In addition to systemic regulation, proteins involved in iron metabolism are produced in small quantities in tissues, including the kidneys, where they may exert either systemic or paracrine functions [10]. Of note, many leukocyte-derived cytokines and liver-derived acute-phase proteins have previously been shown to be associated with the severity of PUUV infection [23–26]. 

Functional iron deficiency during infection or inflammation can result in anaemia. We did not observe an association between low acute-phase haemoglobin levels and ferritin or hepcidin. This finding aligns with the acute-phase response, as elevated levels of ferritin during infection do not necessarily reflect iron stores. The direct correlation between the acute-phase minimum haemoglobin concentration and the convalescence-phase ferritin level suggests that more abundant iron stores are associated with higher haemoglobin levels even during acute PUUV infection. The acute-phase serum creatinine inversely correlated with convalescence-phase iron and transferrin levels. Thus, AKI may impact the availability of iron for haemoglobin synthesis for an extended period after clinical recovery.

LDH, which is characteristically elevated during haemolysis, was increased during the acute phase of PUUV infection. The finding that LDH did not correlate with either minimum haemoglobin or haptoglobin values indicates that significant haemolysis during PUUV infection was unlikely. During PUUV infection, patients often show elevated liver enzymes [27]. As LDH is frequently elevated simultaneously with transaminases, increased LDH concentration may reflect liver damage during PUUV infection.

Serum ferritin and hepcidin correlated with maximum leukocyte count, maximum CRP concentration, minimum platelet count, and length of hospital stay. This is consistent with earlier findings on disease severity indices during haemorrhagic viral infections. In dengue virus infection [5], ferritin is associated with immune activation and coagulation disturbances [5, 9]. In Crimean-Congo haemorrhagic fever, ferritin has been proposed as a potential biomarker for estimating disease prognosis [7, 8], and in severe fever with thrombocytopenia syndrome, changes in hepcidin and ferritin have been linked to fatal outcomes [28, 29]. However, it is uncertain whether the association detected between iron parameters and disease severity represents a pathophysiological connection between iron parameters and disease outcome, or is merely an association with acute phase reactants and more severe disease [10, 30, 31]. Of interest is our observation that the CRP concentration in the acute phase correlates inversely with the NGAL concentration in the convalescent phase. We have previously speculated that CRP may be protective in PUUV-induced AKI [32, 33], but the mechanism underlying this association requires further investigation. The positive correlation of acute phase minimum platelet count and convalescence phase NGAL is somewhat unexpected. A higher minimum platelet count is indicative of milder PUUV infection, whereas elevated NGAL levels are usually associated with delayed recovery from AKI. However, as NGAL is derived from neutrophils, it may also reflect the extent of inflammation. This observation warrants further investigation.

Iron parameters, maximum serum creatinine and NGAL correlated with weight change. Weight gain during acute kidney injury (AKI) typically results from reduced urine output. In PUUV infection, however, the situation is more complex. Increased capillary permeability and fluid extravasation are characteristic features of the infection, often leading to hypotension. This in turn may prompt fluid resuscitation, further contributing to fluid accumulation. Inflammatory responses exacerbate capillary leakage, as demonstrated in our study by Tervo et al. [33] During the recovery phase, polyuria is commonly observed. Therefore, the weight gain seen in acute PUUV infection is likely attributable to a combination of AKI-related oliguria and virus-induced capillary leakage.

Ferroptosis is a form of programmed cell death in which the key initiator is the accumulation of free iron. It is characterised by either the accumulation of lipid-reactive oxygen species (ROS) or the depletion of cellular antioxidants, leading to increased levels of intracellular iron and the deposition of lipid ROS [34]. Recently, ferroptosis has been linked to both AKI and the transition of AKI to CKD. NGAL is a known marker of kidney injury, but it is also an iron-binding protein that harvests free iron from microbes, thereby preventing iron from being used by them [35–37]. In our study NGAL correlated with maximum serum creatinine concentration and maximum leukocyte count. We did not detect a correlation between the severity of AKI (maximum serum creatinine concentration or NGAL) and serum ferritin or hepcidin, but NGAL and serum iron concentration showed a negative correlation. Whether this association relates to the status of oxidative stress during AKI warrants further studies.

Contradictory results have been reported regarding the role of ferritin in AKI. In patients treated in an intensive care unit, elevated serum ferritin levels predicted higher mortality in those with AKI. However, the underlying diagnoses leading to intensive care admission were not reported, making it possible that many of these patients had infections or inflammatory conditions that contributed to elevated ferritin levels [38]. On the other hand, renal failure is more common after cardiopulmonary bypass surgery in patients with lower ferritin levels, possibly due to a limited ability to bind free iron generated by haemolysis during surgery [39]. As all patients were electively scheduled for coronary artery bypass or valvular replacement surgery, their ferritin levels likely reflected iron stores rather than an acute-phase reaction [39]. Notably, elevated serum ferritin levels have also been found to predict recovery of renal function after AKI [40]. 

The relationship between ferritin in AKI is further complicated by the fact that ferritin is composed of 24 subunits of two types: ferritin heavy chain (FtH) and light chain (FtL). FtH possesses ferroxidase activity, which is necessary for iron incorporation into ferritin, thus limiting its toxicity. FtH may also limit calcification and osteoblastic differentiation of smooth muscle cells and promote angiogenesis by binding to high molecular weight kininogen (HKa) [41, 42]. Ferritin binding to HKa could inhibit kallikrein-mediated bradykinin release, thereby limiting the inflammatory response and possibly preventing hypotension [43]. FtH may also influence fibrosis after rhabdomyolysis mediated AKI [44]. In our study, an association between ferritin and blood pressure was not observed.

Injection of lipopolysaccharides induces a prominent renal proteome change in mice, including upregulation of many acute-phase reactants involved in iron homeostasis, namely NGAL, haptoglobin, hemopexin, as well as complement C3 and fibrinogen [45]. If a similar change occurs during infections, measuring local renal production of FtH and FtL, rather than systemic, circulating levels of total serum ferritin, may be necessary to explore the role of iron metabolism in the development of AKI.

In conclusion, we have shown that parameters of iron metabolism undergo distinct changes during PUUV infection, and that iron metabolism was associated with markers of disease severity, such as blood leukocyte and platelet levels, and the length of hospital stay. However, we did not observe an association between iron metabolism and the severity of AKI during PUUV infection.

## Data Availability

Owing to the sensitive nature of the data supporting the findings of this study, the data cannot be shared publicly for ethical reasons. The datasets used and/or analyzed in the current study are available from the corresponding author upon reasonable request.
